# Mycoplasma pneumoniae: not a typical respiratory pathogen

**DOI:** 10.1099/jmm.0.001910

**Published:** 2024-10-30

**Authors:** Richard S. Rowlands, Patrick M. Meyer Sauteur, Michael L. Beeton, On behalf of the ESCMID Study Group for Mycoplasma and Chlamydia Infections (ESGMAC)

**Affiliations:** 1 Microbiology and Infection Research Group, Department of Biomedical Sciences, Cardiff Metropolitan University, Western Avenue, Cardiff. CF5 2YB, UK; 2 Division of Infectious Diseases and Hospital Epidemiology, Children’s Research Center, University Children’s Hospital Zurich, University of Zurich, Zurich, Switzerland

**Keywords:** adhesion, atypical pneumonia, CARDS toxin, extrapulmonary disease, macrolides, P1 type, vaccine-enhanced disease

## Abstract

*Mycoplasma pneumoniae* is a leading cause of community-acquired pneumonia among school-aged children and young adults. Infections occur throughout the year but tend to surge during winter months across Europe. A characteristic epidemic cycle, where a substantial surge in the number of infections occurs, is seen approximately every 1–4 years and hypothesized to be driven by changes in immunity and a shift in circulating variants. Once thought to be an organism of low virulence, it has now been found to possess several virulence factors, including toxin production, biofilm formation and evasion of antibody-mediated immunity. The lack of a cell wall and reduced metabolic pathways limit the options for antibiotic treatment. Acquired macrolide resistance is a growing concern, with >80% of cases in China being macrolide-resistant. Although efforts have been made to develop a vaccine, there are still substantial hurdles to overcome in relation to vaccine-enhanced disease, which results from an inappropriate immune response among vaccinated individuals.

## Graphical abstract

(a). The classic ‘fried-egg’ colonies are commonly seen with the growth of *Mycoplasma pneumoniae* on agar. (b). Scanning electron micrograph of *M. pneumoniae* demonstrates spindle-shaped cells with attachment organelles. *M. pneumoniae* is equipped with a repertoire of virulence factors, which include the production of the community-acquired respiratory distress syndrome toxin, formation of H_2_0_2_ and H_2_S, the immunoglobulin-binding protein of *Mycoplasma*, adhesion and antigenic variability via the P1 adhesin and associated surface proteins and the ability to form biofilms on surfaces.

## Historical perspective

*Mycoplasma pneumoniae* was first isolated in 1944 from the sputum of a patient with atypical pneumonia [1]. Initially referred to as the Eaton agent and thought to be viral due to its ability to pass through a bacteria-retaining filter and its resistance to penicillin and sulphonamides, *M. pneumoniae* later became recognized as a bacterial agent in the 1960s [2]. It was first cultured on a cell-free medium and classified as *M. pneumoniae* in 1963 by Chanock *et al.* [3]. As with all *Mycoplasma* spp., the term mycoplasma is derived from the Greek words ‘mykes’, meaning fungus, and ‘plasma’, meaning formed.

## Clinical presentation

*M. pneumoniae* infects exclusively humans where it primarily causes mild and self-limiting respiratory tract infections. It is responsible for up to 20% of community-acquired pneumonia (CAP) cases in adults and up to 40% of cases in children [4]. Extrapulmonary manifestations can affect almost all organs. For example, severe neurological manifestations such as encephalitis can occur in 7% of children with *M. pneumoniae* infection [5]. Skin manifestations are common, with 25% of patients presenting with non-specific exanthema, urticaria and a *M. pneumoniae*-induced rash and mucositis [6]. Long-term sequelae, including neurological, hepatic, cardiac and renal complications, can occur but are relatively rare.

## Microbial characteristics

### Phenotypic features

*M. pneumoniae* is characterized by its small cellular size (1–2 µm long and 0.1–0.2 wide), absence of a cell wall and essential requirement for sterols [4]. The bacterium exhibits gliding motility and has a characteristic spindle shape with a prominent attachment organelle. Glucose utilization is the primary substrate for energy production [7].

### Genotypic features

The genome of *M. pneumoniae* is ~0.8 Mbp, with a G+C content of 40%, and is regarded as extraordinarily stable, with a lack of evidence for horizontal gene transfer [8]. Various molecular methods are available for the typing of *M. pneumoniae,* which include P1 typing, multilocus variable-number tandem-repeat analysis (MLVA) and multilocus sequence typing (MLST) [9]. P1 typing relies on amplifying RepMP2/3 and RepMP4 containing the region of the *p1* gene (MPN141), with subsequent restriction fragment length polymorphism analysis or Sanger sequencing [10]. Although this method does not give as granular detail as MLVA or MLST, it allows for the differentiation of the broad *p1* type 1 and *p1* type 2 subtypes. Switching between these subtypes has been hypothesized to be one driver for epidemic surges [10,11].

## Laboratory confirmation and safety

### Specimen type

*M. pneumoniae* can be detected in respiratory specimens of patients with respiratory tract symptoms. Sample types can include pharyngeal and nasopharyngeal swabs for detection in the upper respiratory tract; sputum, tracheal aspirates, bronchoalveolar lavage and pleural fluid for detection in the lower respiratory tract and cerebrospinal fluid for evaluating disseminated infection [12]. However, *M. pneumoniae* can be found in the upper respiratory tract of 3–58% of asymptomatic individuals (mainly children), depending on the presence or absence of an epidemic [13].

### Laboratory confirmation

Due to the fastidious growth requirements and slow growth rate (1–3 weeks for positive culture), culture-based methods are uncommon and are typically undertaken for reference or research purposes. If culture is undertaken, broth containing animal serum is required, typically containing a pH indicator for confirmation of acidification of the broth in the absence of turbidity. Growth on agar requires incubation with 5% CO_2_ and the use of a stereomicroscope for observation of small ‘fried egg’ colonies. The lack of a cell wall renders the classic Gram stain method redundant for laboratory confirmation. For these reasons, nucleic acid amplification tests are most common in diagnostic laboratories (‘new gold standard’). These are either commercial or developed in-house and run as a standalone assay or as a multiplex with other respiratory pathogens [14]. The detection of specific antibodies in serum by enzyme immunoassay is not recommended for acute clinical management.

## Laboratory safety

*M. pneumoniae* is considered a hazard group 2 organism by the UK Health and Safety Executive. Handling of samples should be undertaken within containment at biosafety level 2/containment level unless there is suspicion of a pathogen of a greater hazard grouping status. Due to the potential for respiratory tract infection, samples containing viable organisms should be handled within a class II Biological Safety Cabinet.

## Treatment and resistance

### Treatment

As *M. pneumoniae* lacks a cell wall, it is resistant to antibiotics that inhibit cell wall synthesis, such as β-lactam antibiotics. Current National Institute for Health and Care Excellence guidelines recommend clarithromycin for CAP where an atypical pathogen, such as *M. pneumoniae*, is suspected [15], although the effectiveness of macrolides in children with *Mycoplasma* pneumonia remains unclear due to the current lack of clinical trial data. Tetracycline and fluoroquinolone antibiotics show good activity *in vitro* and may be considered, although these classes of antibiotics may have potential toxicities in young children. Doxycycline and levofloxacin or moxifloxacin can be used in children of >7 years of age and in adolescents with skeletal maturity, respectively, according to the Infectious Diseases Society of America. There is an indication that doxycycline can also be administered safely in children <8 years of age for shorter durations (≤21 days) [16]. High levels of acquired macrolide resistance in countries such as Japan have resulted in a shift from prescribing macrolides to the fluoroquinolone tosufloxacin [10]. *M. pneumoniae* infections can be mild and self-limiting [17], which supports the hypothesis of an immune-mediated pathogenesis and should be taken into consideration [18,19]. Corticosteroids and intravenous immunoglobulin, in combination with appropriate antimicrobials, have shown promise in treating refractory *M. pneumoniae* pneumonia [20].

## Resistance

A lack of cell wall bestows an intrinsic resistance to β-lactam and glycopeptide antibiotics. Sulphonamides, trimethoprim, polymyxins, nalidixic acid and rifampicin are also inactive [21]. Acquired resistance resulting in macrolide-resistant *M. pneumoniae* is a growing concern. Resistance is mediated predominantly via point mutations within domain V of the 23S rRNA at position 2063 or 2064 (2058 and 2059 *Escherichia coli* numbering), preventing drug binding. These mutations are consistently found in more than 80–90% of *M. pneumoniae* in China and ~10% in Europe [22]. Clinical and Laboratory Standards Institute methods, and breakpoint values for resistance, have been established for *M. pneumoniae* [23], but due to the slow and fastidious growth requirements, the determination of resistance is primarily by PCR and Sanger sequencing. To date, no tetracycline or fluoroquinolone resistance has been identified from a clinical specimen, although *in vitro* selection has generated isolates with fluoroquinolone resistance and reduced susceptibilities to tetracycline [24,25].

## Pathogenic strategies

### Host range

The host range of natural infection is limited to humans.

### Vectors and sylvatic cycle

No vectors or sylvatic cycle has been documented.

### Virulence factors

*M. pneumoniae* was once thought to be a pathogen of low virulence. However, several virulence factors have been identified in * M. pneumoniae* aiding in colonization and infection. A specialized attachment organelle, layered with a battery of adhesins, firmly anchors the bacterium to host cells, overcoming host clearance processes and aiding colonization.

Several cytotoxic molecules are produced including H_2_O_2_, H_2_S and the community-acquired respiratory distress syndrome (CARDS) toxin [26]. Encoded as a single polypeptide, the CARDS toxin is a vacuolating and ADP-ribosyltransferase exotoxin that binds cell surface receptors, including surfactant protein A and annexin A2, causing cytopathology [27]. The CARDS toxin induces an immunogenic response in a dose-, temperature- and time-dependant manner involving IL-1β, with expression significantly increased following adhesion to host cells [28].

Biofilm formation is a critical aspect of *M. pneumoniae* virulence. P1 type 2 strains show more prolific biofilms than type 1, possibly due to increased *p1* expression [29]. The ability to form biofilms aids in persistence, and studies show that expression levels of CARDS toxin decrease as biofilms evolve and mature, increasing the likelihood of immune evasion [26].

To evade antibody-mediated clearance, *M. pneumoniae* encodes the immunoglobulin-binding protein of *Mycoplasma* [30]. This is a cell surface-localized protein that strongly binds IgG, IgA and IgM and shares homology with the closely related protein M of *Mycoplasma genitalium* [31].

Past studies have proposed differences in virulence between *p1* type 1 and *p1* type 2 isolates based on genomic and biofilm analysis [29,32]. The major flaws in these studies have been the inclusion of a small number of representative strains of each *p1* type and, therefore, the lack of power to make clear differences between the two.

## Epidemiology

### Transmission

Transmission is predominantly via respiratory droplets during periods of close contact with *M. pneumoniae*-positive individuals with a long incubation period of several weeks. As *Mycoplasma* species are prone to desiccation due to the absence of a cell wall, transmission via fomites is likely less of a concern.

### Infection

The *R*_0_ value is predicted to be relatively low at 1.7 (95% confidence interval 1.6–1.9), suggesting low transmissibility [33]. Due to the need for close and continued contact for infection, outbreaks have been documented to occur in closed communities and educational settings [34,36].

### Epidemiology

Endemic infection has been demonstrated throughout the year with surges in infection occurring over the winter months across Europe, along with many other respiratory tract infections. Prior to the COVID-19 pandemic, a cyclical epidemic pattern appeared approximately every 1–4 years [37]. This surge in infection is hypothesized to be driven by waning herd immunity within a population and potential changes in the circulating *p1* type over time [10]. Most epidemiological data come from studies undertaken in Europe, the USA, China and Japan, with a significant lack of data from Central and South America, the African continent and Southeast Asia [38]. This lack of data may reflect the cost and infrastructure required to detect and characterize *M. pneumoniae,* and it serves to highlight the need to develop improved low-cost diagnostic methods to better understand the epidemiology in some of these regions. The introduction of non-pharmaceutical interventions (NPIs) in response to the COVID-19 pandemic substantially disrupted endemic infection, with global detections at an unprecedented low level [39]. With the relaxation of NPIs in the following years, infection remained absent while other respiratory pathogens re-emerged [40,41]. During the winter of 2023/2024, *M. pneumoniae* finally re-emerged across the northern hemisphere [42,45].

### Risk groups

Infection can occur in any age group, although it is predominantly seen in children older than 5 years of age and young adults [37,45]. A study found that *M .pneumoniae* is considered to be the second most prevalent pathogen associated with acute asthma exacerbations in children aged 6–17 years [46], although other studies contradict these findings [47].

### Prevention

Currently, no vaccine is available. Vaccine development has been hampered by vaccine-enhanced disease (VED), in which research participants challenged with formalin-inactivated or live-attenuated strains exhibit more severe clinical symptoms compared with placebo controls [48]. Although the mechanism is not fully understood, lipid-associated membrane proteins are thought to be the driving force behind this observation, which supports the hypothesis of an immune-mediated pathogenesis [49,50]. NPIs, such as social distancing, use of masks, stay-at-home orders, improved hand hygiene and travel restrictions, implemented during the COVID-19 pandemic significantly reduced the number of detections globally in March 2020 [39].

## Open questions

1. What is the major driver for epidemic seasonality?
2. Are macrolides necessary for treating *M. pneumoniae* CAP?
3. Why do some patients develop extrapulmonary complications whereas others do not?
4. Is it possible to develop a vaccine without VED?
5. How long will it be until the first fluoroquinolone-resistant or tetracycline-resistant *M. pneumoniae* is detected in a clinical sample?

## Abbreviations

CAP: community-acquired pneumonia
CARDS: community-acquired respiratory distress syndrome
CLSI: Clinical and Laboratory Standards Institute
LAMPs: lipid-associated membrane proteins
MIRM: mycoplasma-induced rash and mucositis
MLST: multi-locus sequence typing
MLVA: multi-locus variable-number tandem-repeat analysis
VED: vaccine-enhanced disease
